# Higher nicotine dependence and greater smoking abstinence in parental than non-parental smokers: a secondary analysis of smoking cessation trials

**DOI:** 10.3389/fpubh.2025.1687893

**Published:** 2025-11-03

**Authors:** Yiran Ge, Mengyao Li, Tzu Tsun Luk, Derek Yee Tak Cheung, Ningyuan Guo, Henry Sau Chai Tong, Vienna Wan Yin Lai, Sophia Siu Chee Chan, Man Ping Wang, Shengzhi Zhao

**Affiliations:** ^1^School of Nursing, The University of Hong Kong, Pokfulam, Hong Kong SAR, China; ^2^Alice Lee Centre for Nursing Studies, National University of Singapore, Singapore, Singapore; ^3^School of Nursing, Shanghai Jiao Tong University, Shanghai, China; ^4^Hong Kong Council on Smoking and Health, Wan Chai District, Hong Kong SAR, China

**Keywords:** parental smokers, smoking cessation, smoking behaviors, nicotine dependence, community-based intervention, behavioral interventions

## Abstract

**Objective:**

Parental smokers account for approximately one-third of smokers worldwide and often exhibit distinct smoking and quitting behaviors compared to non-parental smokers. Understanding these differences is crucial for developing targeted cessation interventions; however, current evidence remains limited.

**Methods:**

This secondary analysis pooled individual participant data from 10 community-based smoking cessation trials conducted in Hong Kong between 2010 and 2020 (*N* = 10,871 adult daily smokers). We compared parental smokers (those with at least one child) and non-parental smokers in terms of sociodemographic characteristics, smoking behaviors, nicotine dependence (Heaviness of Smoking Index), quitting motivation, and cessation outcomes at 6 months post-intervention. Outcomes included biochemically validated abstinence (exhaled carbon monoxide <4 ppm and salivary cotinine <10 ng/mL) and self-reported 7-day point-prevalence abstinence (PPA). Multivariable regression models were adjusted for age, sex, education, income, and trial year.

**Results:**

Of the participants, 42.2% were parental smokers, who were older and had lower education and income (all *p* < 0.001). They had higher daily cigarette consumption (mean: 14.8 vs. 12.9, adjusted *β* = 1.59, *p* = 0.004) and higher nicotine dependence (9.2% vs. 5.9%, AOR = 1.36, *p* < 0.001). A large number of parental smokers had past quit attempts (61.8% vs. 54.0%, AOR = 1.25, *p* < 0.001) and intentions to quit within 30 days (61.2% vs. 46.4%, AOR = 1.31, *p* < 0.001). At 6 months, parental smokers showed higher validated abstinence (7.7% vs. 5.9%, AOR = 1.37, *p* < 0.001) and self-reported 7-day PPA (15.6% vs. 13.9%, AOR = 1.21, *p* = 0.002). Among parental smokers, those co-living with children had greater abstinence than those not necessarily co-living, for both self-reported 7-day PPA (AOR = 1.43, 95% CI 1.03–1.98, *p* = 0.032) and validated abstinence (AOR = 1.62, 95% CI 1.04–2.52, *p* = 0.032).

**Conclusion:**

Parental smokers showed higher nicotine dependence but greater motivation and higher abstinence rates following brief community-based interventions. Tailored programs should address their elevated addiction while leveraging their motivation to enhance cessation success.

## 1 Introduction

Approximately 33% of smokers worldwide are parental smokers (1). Becoming a parent often serves as a pivotal moment to reconsider smoking behaviors. Research has reported that parental smokers often display greater motivation to quit smoking, driven by a heightened sense of responsibility for their children’s health (2). Consequently, smokers, especially those co-living with children, demonstrate a higher intention to quit and have made more quitting attempts than those without children (3). In the United States, 40% of parental smokers have made at least one quit attempt after childbirth (4). Additionally, parents are more likely to set positive examples of healthy lifestyles for their children, which leads to the perceived higher importance of cessation and prompts sustained efforts to quit (5).

Although parental smokers may display greater motivation to quit smoking, the extent of their success in achieving cessation remains inconclusive. Studies found that greater readiness to quit may predict higher success rates in smoking cessation (6, 7), but parental smokers face challenges in both quitting and maintaining abstinence, resulting in higher relapse rates (8). Moreover, half of the maternal smokers who quit smoking during pregnancy relapsed at 6 months postpartum (9–11). This discrepancy between high motivation to quit and low sustained abstinence rates among parental smokers suggests distinct smoking behaviors and challenges in quitting compared to the general smoking population.

Social cognitive theory (SCT) (12) posits that individuals become increasingly aware of the health risks associated with tobacco use (e.g., secondhand smoke exposure and behavioral modeling) upon becoming parents. This heightened awareness may generate a stronger intention to quit smoking. Parental smokers may demonstrate their commitment to protecting their children’s wellbeing by achieving abstinence, underscoring the concept of parenthood as a “teachable moment” for smoking cessation. The effectiveness of such behavioral changes is further enhanced when parents receive tailored interventions designed to support cessation efforts.

Previous smoking cessation interventions for parents have largely centered on pregnant women (13–16) and expectant fathers (17, 18), which may be due to the vulnerability of fetal and infant health to secondhand smoke exposure (19, 20). Behavioral interventions [such as brief counseling (21, 22), toll-free helplines (5, 23), and mobile programs via short message service (SMS) (24)], as well as pharmacological methods such as nicotine replacement therapy (25) and bupropion (26), have shown effectiveness in aiding cessation. However, the comparative effectiveness of these approaches between parental and non-parental smokers remains uncertain. Identifying the relative effectiveness of different cessation interventions becomes imperative to formulate tailored interventions aimed at supporting parental smokers in their cessation journey.

This study examined differences in smoking characteristics and cessation outcomes between smokers with and without children in Hong Kong. We further examined how residential status (whether parents co-live with their children), number of children, sex of parental smokers, and whether they received financial incentive might affect smoking behavior, considering differences in the perceived risk of tobacco smoke exposure. Additionally, cessation outcomes were explored according to the type of behavioral interventions received.

## 2 Methods

### 2.1 Participant and procedure

This is a prospective secondary analysis of data from 10 randomized controlled trials (RCTs) nested within Hong Kong’s annual “Quit-to-Win” (QTW) community campaign (2010–2021; no trials in 2011, and parental data not collected in 2014). Each trial was registered with ClinicalTrials.gov and followed the guidelines for strengthening the reporting of observational studies in epidemiology (STROBE).

At each annual Quit-to-Win Contest (see Supplementary Table S1; Supplementary Figure S1), participants were recruited at community hotspots (e.g., transport hubs, streets of urban business areas, and shopping malls) across all 18 districts of Hong Kong. Trained smoking cessation (SC) advisors approached potential participants, enrolling Chinese-speaking residents aged 18 years or above who smoked at least one cigarette daily in the past 3 months (verified by exhaled carbon monoxide ≥4 ppm). Eligible individuals were then randomly allocated to intervention or control arms. Smokers using SC medication or other SC services, or who were physically or mentally unable to communicate, were excluded. Deidentified baseline data from all 10,871 RCT participants from 2010 to 2021 were included in the analyses, regardless of treatment allocation. Data were collected after obtaining written consent for participation.

### 2.2 Measures

#### 2.2.1 Sociodemographic characteristics

Sociodemographic characteristics were collected as categorical variables, including sex, age group, educational attainment, marital status, having children (aged<18) or not, number of children, and monthly household income. For data collection from 2010 to 2016, parental smokers were defined as individuals who reported having children, as information on co-residence with children was not collected during this period. From 2017 to 2021, smokers with children were asked whether they lived with their children in the same household, allowing for a more specific classification of parental smokers as those residing with their children. Subgroup analyses were conducted to assess differences in the associations between smoking and quitting characteristics among parental smokers, stratified by residential status, number of children, sex, and receipt of financial incentive.

### 2.3 Smoking and quitting characteristics

#### 2.3.1 Nicotine dependence

Daily cigarette consumption was assessed as the average number of cigarettes smoked per day (CPD) over the past 7 days and subsequently categorized into four groups: “10 or fewer,” “11–20,” “21–30,” and “31 or more,” based on the Heaviness of Smoking Index (HSI) categorization. Time to first cigarette after waking was categorized into “>60 min,” “31–60 min,” “6–30 min,” and “<5 min.” Categorized CPD and time to first cigarette were then combined to calculate the HSI score (range: 0–6), with a score of 5–6 indicating a high level of nicotine dependence.

#### 2.3.2 Motivation to quit

Past quit attempts were assessed using the question “When was the last time you abstained from smoking for 24 h or longer when trying to quit?” Responses were dichotomized into “ever attempted” and “never attempted.”

Intention to quit was assessed by asking, “When will you intend to quit smoking?,” with responses “within 7 days,” “within 30 days,” “within 60 days,” and “undecided.” Perceptions of quitting were assessed using perceived importance, confidence, and difficulty to quit on a scale of 0 (not at all) to 10 (very).

#### 2.3.3 Smoking abstinence outcomes

Smoking abstinence outcomes measured at 6-month follow-up included biochemically validated abstinence, self-reported 7-day point-prevalent abstinence (PPA), smoking reduction by at least 50%, and the accumulated number of quitting attempts. Biochemical validation of abstinence was performed using exhaled carbon monoxide testing with the piCO Smokerlyzer (< 4 ppm considered indicative of abstinence). Outcomes were classified as a binary variable, “quit” or “not quit.”

Salivary cotinine was assessed using the NicAlert test strip (<10 ng/mL defined as abstinence) and similarly categorized as “quit” or “not quit.” Smoking reduction by at least 50% was defined as a reduction in cigarette consumption by 50% or more compared with baseline and was categorized as “yes” or “no.” The accumulated number of quitting attempts was determined by whether a participant had made a quit attempt in the past 7 days and was categorized as “yes” or “no.”

#### 2.3.4 Community-based interventions

A total of 26 intervention groups from 10 RCTs were included to evaluate the effectiveness of various behavioral smoking cessation interventions versus brief advice as a control on abstinence outcomes (see Supplementary Table S1; Supplementary Figure S1). The results of each trial were published elsewhere.

Interventions were categorized into six types based on their main components: (1) self-help materials (a 12-page cessation booklet); (2) very brief advice (30-s general cessation advice); (3) brief advice (5-min cessation counseling using the Ask, Warn, Advise, Refer, and Do-it-again [AWARD] model); (4) active referral to cessation services (proactively introducing existing cessation services with a referral card and providing participants’ contact details to service providers to arrange clinic visits); (5) short message services (SMS) delivering regular text messages; and (6) personalized cessation support via instant messaging (IM).

Participants in higher-intensity intervention groups (active referral, SMS, and IM) also received self-help materials and brief advice at baseline. All participants, regardless of group assignment, received self-help materials as the basic intervention. Since 2013, a financial incentive of HK$500 (≈US$64) was provided to participants who passed the biochemical validation at both 3- and 6-month follow-ups (total HK$1000 ≈ US $128).

### 2.4 Statistical analyses

Given that the proportion of missing data was < 10%, available case analyses were used for all baseline measures. We used an intention-to-treat approach for smoking cessation outcomes by treating participants with missing data as having no changes in smoking behavior from baseline.

Descriptive statistics were used to compare sociodemographic and smoking characteristics between participants with and without children. Differences in nicotine dependence and abstinence outcomes by co-living status with children were assessed using binary logistic regression models, with all outcome measures dichotomized. Associations between parental status (having children or not) and smoking characteristics (e.g., CPD, time to first cigarette, and nicotine dependence) were analyzed using multinomial logistic regression models, allowing for category-specific odds ratios without assuming proportional odds. An ordinal logistic regression analysis was applied to test for linear trends across ordered categories of smoking characteristics. The associations between self-efficacy of quitting (continuous variables) and parental status were estimated using a linear regression analysis, with beta-coefficients (*β*s) reported. All regression analyses were adjusted for sex, age, educational attainment, year of inclusion, and type of interventions.

Subgroup analyses were conducted to examine the effectiveness of the different smoking cessation interventions on biochemically validated abstinence at the 6-month follow-up, stratified by parental status. These analyses used independent binary logistic regression models adjusted for sex, age, educational attainment, and the predictors of successful quit attempts (i.e., nicotine dependence level, past quit attempt(s) within 1 year, and intention to quit within 60 days at baseline). Within the parental smoker group, additional subgroup analysis explored whether quit outcomes varied by co-living status with children, number of children, sex of the parental smoker, and whether they received a financial incentive. All analyses were conducted in Stata/MP version 16.1 (StataCorp, United States) with statistical significance set at a *p*-value of < 0.05.

## 3 Results

### 3.1 Baseline characteristics

Among 10,871 participants, 8,500 (78.2%) completed the 6-month follow-up, with similar retention rates between parental (78.5%) and non-parental smokers (77.9%). Table 1 shows that 42.2% were parental smokers. Compared with non-parental smokers, parental smokers were older, had lower educational attainment, and had lower income (all *p* < 0.001).

**Table 1:** Sociodemographic characteristics of smokers by whether they have a child (*N* = 10,871).

Characteristics	Having at least one child (*n* = 4,586, 42.2%)	No child (*n* = 6,285, 57.8%)	*p*-value
Sex			0.007
Male	3,730 (81.3)	4,982 (79.3)	
Female	855 (18.6)	1,302 (20.7)	
*Missing*	1 (0.02)	1 (0.02)	
Age, years^a^			<0.001
18–39	242 (5.3)	2,156 (34.3)	
40–59	3,246 (70.8)	3,059 (48.7)	
60 or above	1,049 (22.9)	755 (12.0)	
*Missing*	49 (1.1)	315 (5.0)	
Marital status^a^			<0.001
Single	204 (4.4)	3,455 (55.0)	
Married/co-habited	4,082 (89.0)	1845 (29.4)	
Divorced/widowed	263 (5.7)	284 (4.5)	
*Missing*	37 (0.8)	701 (11.2)	
Co-live with child (aged < 18)			N. A.
None	-	5,070 (80.7)	
One child	2,189 (47.7)	-	
Two children	1,677 (36.6)	-	
Three or more children	720 (15.7)	-	
*Missing*	0 (0.0)	1,215 (19.3)	
Educational attainment^a^			<0.001
Primary or below	821 (17.9)	376 (6.0)	
Secondary	2,943 (64.2)	3,364 (53.5)	
Tertiary or above	605 (13.2)	1,565 (24.9)	
*Missing*	217 (4.7)	980 (15.6)	
Monthly household income (HK $)^a^			0.272
< 10,000	1,282 (28.0)	1,469 (23.4)	
10,000–19,999	1,498 (32.7)	1810 (28.8)	
20,000–29,999	674 (14.7)	808 (12.9)	
30,000–39,999	305 (6.7)	310 (4.9)	
≥ 40,000	262 (5.7)	284 (4.5)	
*Missing*	565 (12.3)	1,604 (25.5)	

Data are n (%) unless otherwise stated. HSI, Heaviness of Smoking Index. *p* generated by the chi-squared test. US$ 1 = HK$ 7.8.

a Sample size varied due to missing values.

### 3.2 Smoking and quitting characteristics among smokers with and without children

Table 2 shows that parental smokers had higher cigarette consumption (mean 14.8 (9.5) vs. 12.9(8.9), adjusted *β* = 1.59, *p* = 0.004), were more likely to smoke immediately after awakening (≤5 min: 41.9% vs. 35.5%, adjusted odds ratio, AOR = 1.26, 95% CI 1.13–1.41, *p* < 0.001), and had higher nicotine dependence (high: 9.2% vs. 5.9%, AOR = 1.36, 95% CI 1.14–1.63, *p* < 0.001).

**Table 2:** Smoking and quitting characteristics by whether having a child (*N* = 10,871).

Indicators	Having at least one child (*n* = 4,586, 42.2%)	No child (*n* = 6,285, 57.8%)	Crude OR/β (95%CI)	*p*-value	Adjusted OR/β * (95%CI)	*p*-value
Daily cigarette consumption (CPD)						
1–10	2028 (44.3)	3,449 (54.9)	1		1	
11–20	2020 (44.2)	2,353 (37.5)	1.46 (1.35, 1.58)	<0.001	1.20 (1.09, 1.32)	<0.001
21–30	331 (7.2)	261 (4.2)	2.16 (1.82, 2.56)	<0.001	1.56 (1.28, 1.91)	<0.001
>30	196 (4.3)	215 (3.4)	1.55 (1.27, 1.90)	<0.001	1.05 (0.83, 1.34)	0.67
*Mean (SD)* *^b^*	14.8 (9.5)	12.9 (8.9)	1.84 (1.48, 2.20)	<0.001	1.59 (0.19, 0.98)	0.004
*P for trend* *^a^*				<0.001		<0.001
Time to first cigarette after awakening						
>60 min	1,004 (22.1)	1,680 (27.2)	1		1	
31–60 min	496 (11.0)	798 (12.9)	1.04 (0.91, 1.19)	0.57	1.10 (0.94, 1.29)	0.21
6–30 min	1,131 (25.0)	1,512 (24.5)	1.25 (1.12, 1.40)	<0.001	1.23 (1.09, 1.40)	<0.001
≤5 min	1900 (41.9)	2,192 (35.5)	1.45 (1.31, 1.60)	<0.001	1.26 (1.13, 1.41)	<0.001
*P for trend* *^a^*				<0.001		<0.001
Nicotine dependency (HSI)						
Low (≤ 2)	1956 (43.2)	3,230 (52.3)	1		1	
Moderate (3–4)	2,151 (47.5)	2,581 (41.8)	1.38 (1.28, 1.49)	<0.001	1.21 (1.10, 1.32)	<0.001
High (5–6)	418 (9.2)	365 (5.9)	1.89 (1.63, 2.20)	<0.001	1.36 (1.14, 1.63)	<0.001
*P for trend* *^a^*				<0.001		<0.001
Past quit attempt(s)						
Never	1737 (38.2)	2,861 (46.0)	1		1	
Ever	2,810 (61.8)	3,361 (54.0)	1.38 (1.27, 1.49)	<0.001	1.25 (1.14, 1.37)	<0.001
Intention to quit						
No intention within 30 days	1762 (38.8)	3,304 (53.6)	1		1	
Intention within 30 days	2,776 (61.2)	2,856 (46.4)	1.82 (1.69, 1.97)	<0.001	1.31 (1.20, 1.44)	<0.001
Perception of quitting mean (SD)^b^						
Importance	7.4 (2.3)	6.8 (2.4)	0.65 (0.56, 0.74)	<0.001	0.41 (0.31, 0.52)	<0.001
Confidence	5.8 (2.4)	5.6 (2.4)	0.23 (0.14, 0.32)	<0.001	0.11 (0.00, 0.22)	0.049
Difficulty	7.0 (2.6)	6.8 (2.6)	0.16 (0.62, 0.26)	<0.001	0.21 (0.09, 0.32)	<0.001
Quitting outcomes at 6 months						
Self-reported 7-day PPA	717 (15.6)	872 (13.9)	1.15 (1.03, 1.28)	0.010	1.21 (1.07, 1.36)	0.002
Validated abstinence	355 (7.7)	372 (5.9)	1.33 (1.15, 1.55)	<0.001	1.37 (1.16, 1.61)	<0.001
Smoking reduction by >50%	2,621 (59.1)	3,315 (55.3)	1.17 (1.08, 1.27)	<0.001	0.89 (0.80, 0.98)	0.013
Quit attempt (accumulated)	2,320 (50.6)	2,581 (41.1)	1.32 (1.20, 1.45)	<0.001	1.18 (1.06, 1.32)	0.002

OR, odds ratio, generated by a multinomial logistic regression model except when specially defined; CI, confidence interval, HSI: Heaviness of Smoking Index; SD, standard deviation. CPD, cigarettes per day. *Adjusted for sex, age of participation, educational attainment, and year of inclusion.

a *p*-value generated by an ordinal logistic regression model.

b β-coefficient generated by a linear regression model.

For quitting intention, parental smokers reported more past quit attempts (61.8% vs. 54.0%, AOR = 1.25, 95% CI 1.14–1.37) and had stronger intention to quit within 30 days (61.2% vs. 46.4%, AOR = 1.31, 95% CI 1.20–1.44). Additionally, parental smokers perceived higher importance (7.4% vs. 6.8%, AOR = 0.41, 95% CI 0.31–0.52) but also higher difficulty (7.0% vs. 6.8%, AOR = 0.21, 95%CI 0.09–0.32) of quitting, as compared to non-parental smokers (all Ps < 0.001).

At 6-month follow-up, parental smokers showed significantly higher validated abstinence (7.7% vs. 5.9%, AOR = 1.37, 95% CI 1.16–1.61, *p* < 0.001), self-reported 7-day PPA (15.6% vs. 13.9%, AOR = 1.21, 95% CI 1.07–1.36, *p* = 0.002), and more accumulated quit attempts (50.6% vs. 41.1%; AOR = 1.18, 95% CI 1.06–1.32, *p* = 0.002).

### 3.3 Subgroup analyses

Table 3 shows that parental smokers who co-live with their children have significantly higher smoking abstinence at 6 months for both self-reported 7-day PPA (18.8% vs. 13.9%, AOR = 1.43, 95% CI 1.03–1.98, *p* = 0.032) and validated abstinence (9.9% vs. 6.5%, AOR = 1.62, 95% CI 1.04–2.52, *p* = 0.032) than those who have children but do not necessarily co-live with children. No significant differences were observed in quitting outcomes between smokers with fewer children vs. with more children, between smoking fathers vs. mothers, and between those that received a financial incentive vs. those that did not (all *p* > 0.05).

**Table 3:** Quitting outcomes for parental smokers by residential status, number of children, sex of parental smokers, and receipt of financial incentive (*N* = 4,586).

Subgroups	Validated abstinence	Self-reported 7-day PPA
Parental smokers		
Having children	192/2941 (6.5)	408/2941 (13.9)
Having and co-living with children	163/1645 (9.9)	309/1645 (18.8)
Adjusted OR* (95% CI)	1.62 (1.04, 2.52)	1.43 (1.03, 1.98)
*p-value*	0.032	0.032
Number of children		
1	158/2189 (7.2)	326/2189 (14.9)
2	146/1677 (8.7)	281/1677 (16.8)
3	51/720 (7.1)	110/720 (15.3)
Adjusted OR* (95% CI)	1.11 (0.94, 1.31)	1.12 (0.99, 1.26)
*p-value*	0.207	0.070
Smoking mother or father		
Smoking father	302/3730 (8.1)	590/3730 (15.8)
Smoking mother	53/855 (6.2)	127/855 (14.9)
Adjusted OR* (95% CI)	0.80 (0.58, 1.09)	0.97 (0.78, 1.21)
*p-value*	0.160	0.810
Received financial incentive		
No	77/1186 (6.5)	167/1186 (14.1)
Yes	278/3400 (8.2)	550/3400 (16.2)
Adjusted OR* (95% CI)	1.28 (0.99, 1.67)	1.18 (0.98, 1.42)
*p-value*	0.062	0.087

OR, odds ratio, generated by binary logistic regression, with “having children, living with one child, smoking father, and not received financial incentive” defined as the reference category. CI, confidence Interval. *Adjusted sex, age, education, nicotine dependence level, past quit attempt(s) within 1 year, and intention to quit in 60 days at baseline.

Table 4 assesses the effectiveness of different interventions on smoking cessation among smokers with and without children. Parental smokers had both higher biochemically validated abstinence (total: 7.7% vs. 5.9%, AOR = 1.37, 95% CI 1.16–1.61, *p* < 0.001) and self-reported abstinence (total: 15.6% vs. 13.9%, AOR = 1.21, 95% CI 1.07–1.36, *p* = 0.002) at 6 months after receiving all types of community-based brief intervention.

**Table 4:** Effect of community-based brief interventions on quitting outcomes at 6 months by whether having a child (*N* = 10,871).

Interventions received	Having at least one child (*n* = 4,586, 42.2%)	No child (*n* = 6,285, 57.8%)	Crude OR (95%CI)	*p*-value	Adjusted OR (95%CI)*	*p*-value
Biochemically validated abstinence						
Very brief advice, *n* = 1915	39 (5.9)	51 (4.1)	1.47 (0.96, 2.26)	0.07	1.13 (0.70, 1.83)	0.62
Brief advice, *n* = 2,265	85 (6.3)	34 (3.7)	1.74 (1.16, 2.62)	0.008	1.54 (0.96, 2.48)	0.074
Self-help materials, *n* = 822	28 (5.6)	7 (2.2)	2.70 (1.16, 6.25)	0.021	2.11 (0.81, 5.45)	0.12
Active referral to cessation services (AR), *n* = 1,360	53 (10.0)	55 (6.6)	1.57 (1.06, 2.33)	0.180	1.43 (0.91, 2.26)	0.12
Text messages (SMS), *n* = 1939	66 (8.5)	80 (6.9)	1.26 (0.90, 1.77)	0.18	1.45 (1.01, 2.07)	0.045
Personalized instant messaging (IM), *n* = 2,570	84 (10.9)	145 (8.1)	1.39 (1.05, 1.85)	0.021	1.29 (0.96, 1.72)	0.089
Total	355 (7.7)	372 (5.9)	1.33 (1.15, 1.55)	<0.001	1.37 (1.16, 1.61)	<0.001
Self-reported abstinence						
Very brief advice, *n* = 1915	95 (14.3)	128 (10.2)	1.47 (1.11, 1.95)	0.008	1.28 (0.91, 1.78)	0.15
Brief advice, *n* = 2,265	176 (13.0)	78 (8.5)	1.61 (1.21, 2.13)	0.001	1.39 (0.99, 1.93)	0.053
Self-help materials, *n* = 822	67 (13.4)	35 (11.1)	1.24 (0.81, 1.91)	0.32	1.39 (0.83, 2.34)	0.21
Active referral to cessation services (AR), *n* = 1,360	99 (18.7)	144 (17.3)	1.10 (0.83, 1.46)	0.52	0.98 (0.71, 1.36)	0.92
Text messages (SMS), *n* = 1939	123 (15.9)	171 (14.7)	1.10 (0.85, 1.41)	0.48	1.32 (1.01, 1.74)	0.041
Personalized instant messaging (IM), *n* = 2,570	157 (20.4)	315 (17.5)	1.20 (0.97, 1.49)	0.087	1.17 (0.94, 1.46)	0.17
Total	717 (15.6)	872 (13.9)	1.15 (1.03, 1.28)	0.010	1.21 (1.07, 1.36)	0.002

OR, odds ratio, generated by binary logistic regression. CI, confidence interval. *Adjusted sex, age, education, nicotine dependence level, past quit attempt(s) within 1 year, and intention to quit in 60 days at baseline.

## 4 Discussion

The present study investigated the impact of parenthood on smoking behavior and cessation outcomes among smokers in Hong Kong. Baseline characteristic analysis revealed that parental smokers are associated with lower income and educational attainment. Several studies have found a similar strong association between parents’ socioeconomic status and indoor smoking prevalence (27, 28). Parental smokers with lower educational attainment often reside in communities with higher smoking acceptance, where people are more inclined to perceive smoking in the presence of children as normal (29, 30). Socially disadvantaged individuals often experience greater daily stress and consequently smoke more cigarettes as a coping mechanism (31), with stress possibly mediating the relationship between socioeconomic status and smoking prevalence (32).

For this study, parenthood was associated with more intensive smoking patterns and higher addiction levels, as evidenced by a greater proportion of parental smokers who smoked over 15 cigarettes per day and smoked within 5 min of awakening. Time to first cigarette is recognized as a stronger predictor of physiological nicotine dependence, whereas the number of cigarettes per day better reflects psychological dependence on nicotine’s negatively reinforcing effects (33). This may suggest that parental smokers have both stronger physiological and psychological nicotine dependence, making them more persistent smokers compared to non-parental smokers. The demands of parenting may increase parental smokers’ reliance on smoking as a coping mechanism for stress, anxiety, or fatigue (34). These factors together make parental smokers more resistant to quitting and more persistent in their smoking behavior than non-parental smokers.

On the other hand, parental smokers demonstrated greater motivation to quit, perceived quitting as more important, and had more successful quitting at 6 months post-intervention. Parenthood is a known motivating factor for smoking cessation as well as a protective factor to deter smoking initiation (5, 35). Parental smokers are more likely to consider their children’s potential adverse health outcomes when making decisions regarding smoking and quitting (2). However, their high nicotine dependence increases the likelihood of relapse, which may explain their more frequent past quit attempts. This finding aligns with prior evidence reporting that smoking fathers with more quit attempts have a higher risk of nicotine dependence (36).

Subgroup analyses showed that parental smokers living with children had more favorable quitting outcomes at 6 months post-intervention compared to parental smokers who do not live with children. The presence of children in the household may enhance the success of smoking cessation efforts among parents, potentially driven by increased concern about secondhand smoke exposure on children’s health. The lack of significant differences in quitting outcomes between smokers with fewer vs. more children could potentially be due to the overarching motivation that parenthood provides for smoking cessation. Parents may be equally motivated to quit to protect their children’s health, regardless of family size. The lack of significant differences in quitting outcomes for smoking mothers and fathers may be explained by the progressive convergence of gender roles, leading to less pronounced differences in smoking patterns between mothers and fathers. The absence of differential effects by financial incentive suggests that intrinsic motivations, such as the desire to protect children from tobacco-related disease, may be equal to or more important than extrinsic rewards in supporting cessation among parental smokers.

Community-based behavioral interventions were found to be effective in supporting smoking cessation among parents. Such brief intervention models provided more convenient ways for parental smokers to access smoking cessation information, counseling, and services. Proactively approaching parental smokers and providing brief advice, a self-help booklet, and active referral for smoking cessation services showed higher cessation outcomes in parental smokers, echoing prior research showing that proactively enrolling parents in smoking quitlines during pediatric visits is associated with greater quitline use compared to mere provider recommendations (37). Cessation programs that effectively deliver assistance to parents who might not otherwise receive it are likely to achieve more favorable outcomes (38). However, to select the most appropriate type of intervention, more information is necessary. This aligns with the CEASE program’s recommendation to consider multiple perspectives when developing and implementing a tailored practice change program (39).

This study has several limitations. First, although the data were derived from 10 RCTs, several design limitations preclude causal inference. The intervention components were not entirely identical across trials, participants were not randomized according to parental status, and the original trials were not designed to evaluate the causal impact of parental status on cessation outcomes. As the study design used a secondary analysis approach, a definitive cause-and-effect relationship could not be established. Second, the generalizability of findings, especially the differences in cessation outcomes by the sex of parental smokers, may be constrained as the study primarily included Chinese-speaking Hong Kong residents. Parenting and smoking norms differ markedly between East Asian societies and the West. In East Asia, smoking is accepted among men, symbolizing masculinity and authority, whereas it is less accepted among women (40). This contrasts with Western societies, with less gender disparity in smoking rates (41, 42). Third, the exclusion of individuals using smoking cessation medication or other services might have introduced bias. Finally, other factors that potentially influence parents’ smoking and cessation behavior, including children’s age and health status or the presence of other smokers in the household (e.g., spouse or cohabitants), were not measured in this study. Future studies examining a comprehensive range of child- and family-related health and smoking behavior are warranted.

## 5 Conclusion

Parental smokers exhibited not only greater nicotine dependence but also stronger motivation to quit than non-parental smokers, and they achieved higher abstinence rates following brief community-based interventions. Tailored cessation strategies that address nicotine dependence and harness parental motivation are warranted to increase cessation rates and inform future tobacco control policy.

## Data Availability

The original contributions presented in the study are included in the article/Supplementary material, further inquiries can be directed to the corresponding author.
