# Supplementary material for: Higher nicotine dependence and greater smoking abstinence in parental than non-parental smokers: a secondary analysis of smoking cessation trials
Source: Front Public Health. 2025 Nov 3;13:1687893. doi: 10.3389/fpubh.2025.1687893 (PMC12620414; doi:10.3389/fpubh.2025.1687893)
Supplement: Supplementary file 1 [file Table_1.DOCX]

**Supplementary Materials**

**Table S1. Contents of the 10 trials included in the study**

| **Year** | **Sample size** | **Trial registry/DOI** | **Intervention groups and components** | | |
| --- | --- | --- | --- | --- | --- |
|  |  |  | **A** | **B** | **C** |
| 2010 | 831  A=441  B= 390 | 10.1007/s11121-017-0823-z | **Brief advice**  5-min Student-led on-site counselling (AWARD)  12-page self-help booklet | **Self-help material**  12-page self-help booklet |  |
| 2012 | 1193  A=265  B=419  C=432 | [NCT01670864](https://clinicaltrials.gov/ct2/show/NCT01670864) | **Brief advice**  5-min Student-led on-site counselling  Student-led telephone booster (1week, 1 month)  Health education card  12-page self-help booklet | **SMS**  SMS (x16, in 4 week, 2 sets based on readiness to quit)  12-page self-help booklet | **Self-help material**  12-page self-help booklet |
| 2013 | 1143  A=379  B=385  C=379 | [NCT01928251](https://clinicaltrials.gov/ct2/show/NCT01928251)/ 10.1016/j.addbeh.2016.11.006 | **Brief advice**  Early informed incentive (1 week, 1month)  AWARD  12-page self-help booklet  Education card | **Brief advice**  Late-informed incentive (3 month)  AWARD  12-page self-help booklet  Education card | **Brief advice**  Un-informed incentive  (given at 6 month)  AWARD  12-page self-help booklet Education card |
| 2015 | 1226  A=402  B=416  C=408 | [NCT02539875](https://clinicaltrials.gov/ct2/show/NCT02539875)/  10.1001/jamainternmed.2017.5793 | **Active referral**  LAR, Referral card  AWARD  Health warning leaflet  12-page self-help booklet | **Brief advice**  AWARD  Health warning leaflet  12-page self-help booklet | **Very brief advice**  30-seconds General smoking cessation advice (VBA)  12-page self-help booklet |
| 2016 | 1163  A=395  B=385  C=383 | [NCT02804880](https://clinicaltrials.gov/ct2/show/NCT02804880)/  10.1111/add.15029 | **Active referral**  HAR (Tel remind 1 week; IM: 3 in 1week & 1 in 2-8 week; attend remind at 7^th^ message), Referral card  AWARD  Warning leaflet (as 2015)  12-page self-help booklet | **SMS**  LTM (IM: 3/week in 1 month & 1/week in 2 month)  AWARD  Referral card  Warning leaflet (as 2015)  12-page self-help booklet | **Very brief advice**  VBA  12-page self-help booklet |
| 2017 | 1185  A=591  B=594 | [NCT03182790](https://clinicaltrials.gov/ct2/show/NCT03182790)/  [10.1016/S2589-7500(19)30082-2](https://doi.org/10.1016/S2589-7500(19)30082-2) | **Instant messaging**  IM semi-personalized messages based on ACT (IMPACT)  AWARD  LAR, Referral card  A5 leaflet  12-page self-help booklet | **Very brief advice**  VBA  12-page self-help booklet |  |
| 2018 | 1097  A=565  B=532 | NCT03565796/ 10.1016/j.lanwpc.2021.100189 | **Active referral**  LAR, Incentive for smoking cessation service use (HK$ 300), Referral card  AWARD  A5 leaflet  12-page self-help booklet | **Very brief advice**  VBA  12-page self-help booklet |  |
| 2019 | 844  A=422  B=422 | NCT03992742 | **Instant messaging**  Personalized instant messaging-based adaptive intervention (PIM-AI)  LAR, Referral card  AWARD  A5 leaflet  12-page self-help booklet | **Instant messaging**  Regular instant messaging-based adaptive intervention (RIM-AI)  LAR, Referral card  AWARD  A5 leaflet  12-page self-help booklet |  |
| 2020 | 1165  A=583  B=582 | NCT04399967/  10.18332/tid/163176 | **Instant messaging**  IM with COVID-19 related health warning  AWARD  A5 leaflet on COVID-19  12-page self-help booklet | **SMS**  SMS (x16, on general health)  AWARD  A5 leaflet  12-page self-help booklet |  |
| 2021 | 1105  A=553  B=552 | NCT04909320 | **Instant messaging**  IM with positive psychology-guided peer support  AWARD  A5 leaflet  12-page self-help booklet | **SMS**  SMS (x4, follow-up reminder)  AWARD  A5 leaflet  12-page self-help booklet |  |

Incentives (HK$ 500, US$ 1 = HK$ 7.8) are provided to participants whose abstinence were biochemically validated since 2013

AWARD: Ask, Warn, Advise, Refer, Do-it-again; SMS: Text messages delivered via Short Message Service; QI: Quit immediately; HAR: Hight intensity and personalized active referral; LAR: Low-intensity active referral; LTM: Low-intensity text messaging; TEL: 5-min brief telephone counselling by a trained nurse; IM: Instant messaging; VBA: Very brief general smoking cessation advice; IMPACT: IM semi-personalized messages based on ACT; PIM-AI: Personalized instant messaging-based adaptive intervention; RIM-AI: Regular instant messaging-based adaptive intervention.
