# Supplementary figures and images for: Higher nicotine dependence and greater smoking abstinence in parental than non-parental smokers: a secondary analysis of smoking cessation trials
Source: Front Public Health. 2025 Nov 3;13:1687893. doi: 10.3389/fpubh.2025.1687893 (PMC12620414; doi:10.3389/fpubh.2025.1687893)

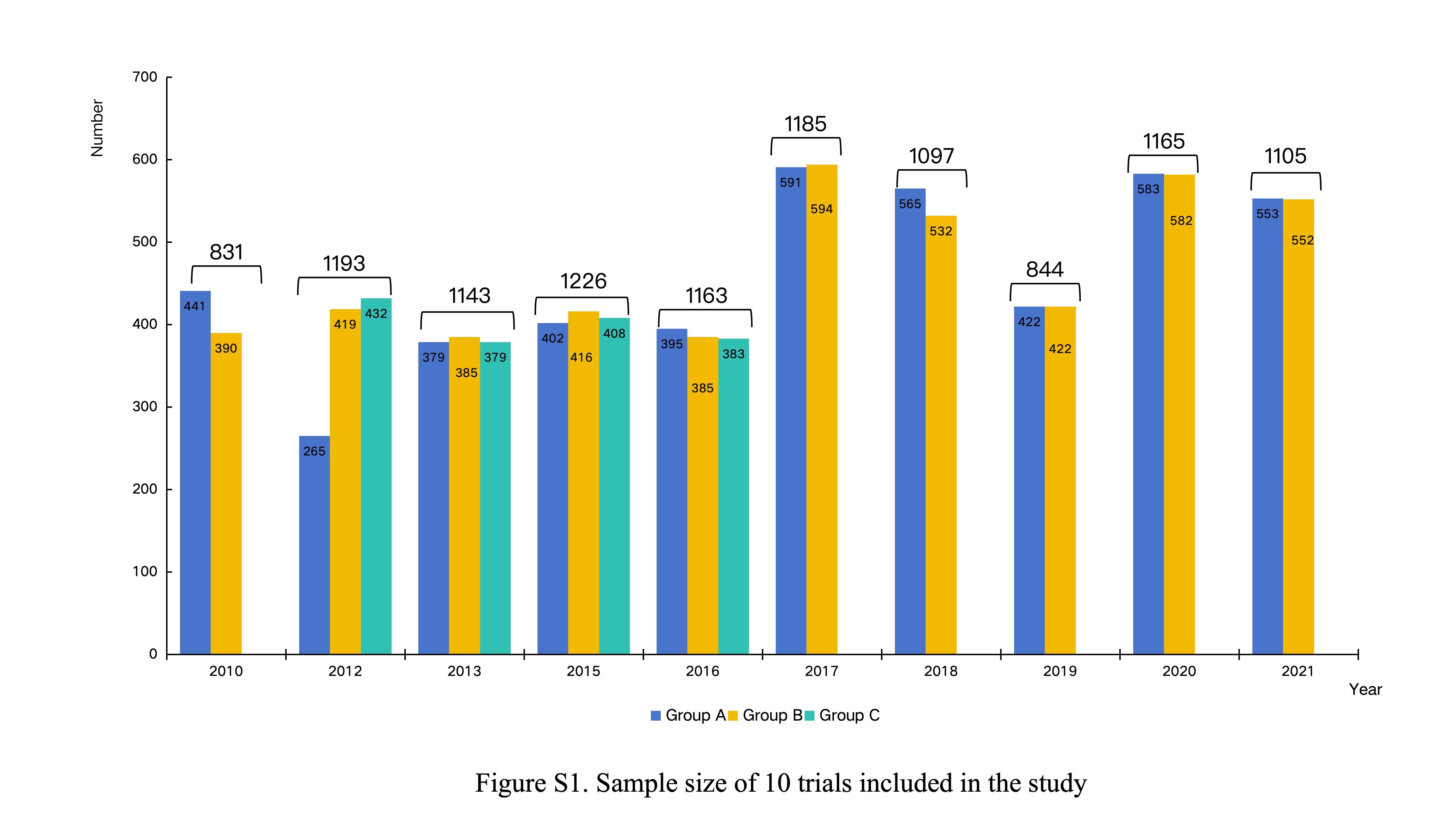

Supplement: Supplementary file 3 [file Figure_1.JPEG]
